# Advances in the study of juvenile hormone signaling

**DOI:** 10.3389/fphys.2026.1788194

**Published:** 2026-04-15

**Authors:** Yue Gao

**Affiliations:** 1 College of Food Science and Technology, Wuhan Business University, Wuhan, China; 2 Guangzhou Key Laboratory of Insect Development Regulation and Application Research, School of Life Sciences, South China Normal University, Guangzhou, China

**Keywords:** insect, intracellular signalling, juvenile hormone, membrane signaling, metamorphosis

## Abstract

Juvenile hormone (JH) and 20-hydroxyecdysone (20E) synergistically regulate the timely onset of metamorphosis during insect metamorphosis. JH regulates multiple physiological processes during insect development via two distinct pathways: intracellular signaling and membrane signaling. Since the discovery of intracellular receptors in *Drosophila melanogaster*, the mechanism of JH intracellular signaling has been well understood, whereas the study of JH membrane signaling has been relatively slow. In recent years, with the optimization of phosphorylated protein detection technology and improvements in sensitivity, the study of JH membrane receptors and the molecular regulation of membrane signaling has made significant progress. In this paper, we provide an overview of JH intracellular and membrane signaling pathways, with a focus on JH membrane signaling, including induced physiological phenomena, potential membrane receptors, downstream phosphorylation cascades, and interactions between JH membrane signaling and hormone signaling.

## 1 Introduction

Juvenile hormone (JH) is an important hormone that regulates metamorphosis and reproduction in insects (Jindra et al., 2013; Jindra et al., 2015). In insect larvae, 20-hydroxyecdysone (20E) is well known to act as a molting hormone to induce molting and metamorphosis, whereas JH inhibits metamorphosis by antagonizing 20E signaling and inhibiting ecdysone synthesis, which together synergistically regulate the timely onset of metamorphosis (Liu S. et al., 2018; Truman and Riddiford, 2019). In many female insects, JH acts as a gonadotrophic hormone stimulating insect vitellogenesis, but ecdysteroids control this process in Diptera and some Hymenoptera and Lepidoptera (Song et al., 2014; Roy et al., 2018; Jiang et al., 2020; Zhu et al., 2020; Luo et al., 2021). In addition, JH plays an important role in the regulation of diapause, fertility, behavior, immunity, and social hierarchy (Jindra et al., 2013; Jindra et al., 2015; Palli, 2023; Barton et al., 2024; Hafeez et al., 2025). Eight major juvenile hormones, JH 0, JH I, JH II, JH III, 4-methyl JH I, JHB3, JHSB3, and methyl farnesoate, are present in insects, of which JHSB3 and JHB3 are synthesized specifically in Hemiptera and Diptera, respectively, JH I and JH II in Lepidoptera, and JH III was present in most insect species, with social insects having generally higher JH III titers (Wen et al., 2015; Nur Aliah et al., 2021; Belles, 2020; Yi et al., 2023). For larva-to-larva molts, a high JH titer is required, as JH works in conjunction with 20E to maintain the juvenile status by preventing metamorphosis. The metamorphic molt takes place when JH titer drops during the final instar (Dubrovsky, 2005).

**Figure 1:**
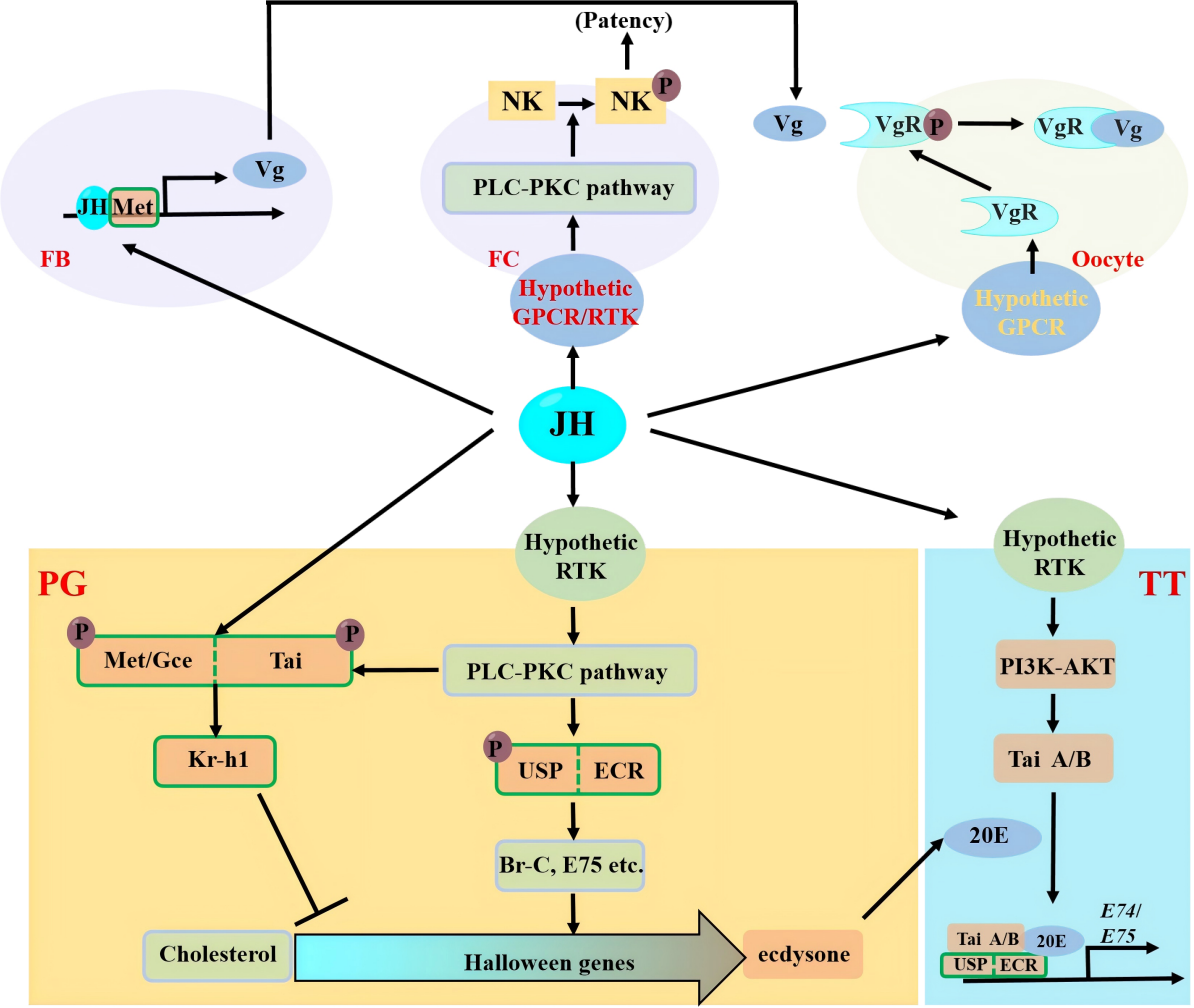
Molecular mechanism of JH intracellular and membrane signaling in regulating development of insects. JH, juvenile hormone; 20E, 20-hydroxyecdysone; GPCR, G Protein-Coupled Receptor; RTK, Receptor Tyrosine Kinase; PLC, Phospholipase C; PI3K, Phosphatidylinositide 3-kinase; AKT, Protein kinase B; FB, Fat body; FC, Follicular cells; NK, Na^+^ K^+^ ATPase; PG, Prothoracic gland; TT, Target tissue.

JH was first discovered and defined by Wigglesworth in *Rhodnius prolixus* in 1934 (Wigglesworth, 1934). The first crude extract of JH was isolated by Williams in 1956 from the abdomen of the male of the moth of the silkworm, *Bombyx mori*. In 1956, Williams first isolated crude JH extracts from the abdomen of male silkworm moths (Williams, 1956). It was not until 1967 that Roller identified JH as a sesquiterpene compound (Roller et al., 1967). With the elucidation of JH’s chemical structure, research into its molecular mechanisms in insects has deepened progressively. Research on JH regulatory mechanisms has now spanned nearly 90 years. The remarkable advancement of molecular biology and genetic experimental techniques has enabled substantial progress in JH studies. This article reviews the current understanding of JH intracellular signaling, membrane signaling-related transmission mechanisms, and action mechanisms, highlighting the functional diversity of JH across different developmental stages and tissues in insects (**Figure 1**).

## 2 JH intracellular receptors - Met/Gce

### 2.1 Met

The JH intracellular receptor gene *Methoprene-tolerant* (*Met*) was discovered by Wilson and Fabian during mutagenesis screening of *Drosophila melanogaster* using ethyl methanesulfonate. Excessive treatment with the JH analog methoprene induces pseudotumor formation in *D. melanogaster* larvae, whereas *Met* mutants exhibit over 100-fold tolerance to methoprene and JH III while inhibiting pseudotumor development (Wilson and Fabian, 1986). Subsequent studies confirmed that compared to wild-type flies, *Met* mutant flies showed no significant differences in JH or JH analog permeability through epidermal cells, excretion, isolation, metabolism, or other insect mechanisms of resistance development (Shemshedini and Wilson, 1990; Willis, 2007). JH-deficient insects exhibit premature pupation or pupal mortality, whereas *Met* mutant fruit flies develop normally into adults with only subtle phenotypes like reduced reproductive capacity. The absence of direct experimental evidence for JH-Met binding led to persistent skepticism among insect researchers regarding Met’s role as an intracellular JH receptor. It was not until 2005 that researchers demonstrated direct binding between Met and JH III using coupled transcription and glucan-coated charcoal methods (Miura et al., 2005), followed by confirmation that Met regulates transcription in a JH-dependent manner via the yeast Gal4-DBD/UAS system (Charles et al., 2011). Subsequently, studies in *Tribolium Castaneum* revealed that treating pupae with Methoprene or JH III within 0–12 hours post-pupation resulted in mortality. However, when the same treatment was applied to house crickets subjected to RNA interference against *Met*, only 2% of the insects died, and metamorphosis was accelerated (Konopova and Jindra, 2007; Palli, 2023).

### 2.2 Gce

Further *D. melanogaster* studies identified the homologous gene *germ-cell expressed* (*Gce*) through gene screening, exhibiting expression patterns similar to *Met*. Double mutant *D. melanogaster Met^27^Gce^2.5K^* exhibited developmental abnormalities resembling those of complete JH-deficient *D. melanogaster*. Genetic experiments confirmed functional redundancy between *Met* and *Gce* (Abdou et al., 2011), resolving the discrepancy between *Met* mutant phenotypes and JH-deficient fly lines. Over three decades of scientific exploration have conclusively established *Met* as the intracellular JH receptor.

### 2.3 Met/Gce complex formation with other molecular chaperones

Met belongs to the bHLH-PAS transcription factor family, containing a bHLH domain for DNA binding, a PAS-A domain for protein interaction, and a PAS-B domain for ligand binding (Ashok et al., 1998). In *D. melanogaster*, JH binding to Met promotes its translocation from the cytoplasm to the nucleus, thereby initiating downstream gene expression (He et al., 2014). In Kc cells, the intact Met protein exhibits subcellular localization in both the nucleus and cytoplasm in the absence of JH treatment. Following JH treatment, Met localizes almost exclusively to the nucleus (Gao et al., 2022a). Met protein fragments containing different domains exhibit distinct subcellular localizations and respond differently to JH treatment. The protein fragment containing the bHLH domain consistently resides in the nucleus, while the fragment containing the PAS-A domain remains localized in the cytoplasm. The fragment containing the PAS-B domain and C-terminal region (Met-PAS-B+) primarily localizes in the cytoplasm in JH-untreated cells. Upon JH treatment, the Met-PAS-B+ protein fragment translocates to the nucleus (Gao et al., 2022a). Upon binding to Met, JH enhances serine phosphorylation at position 219 of the JH-induced chaperone protein Hsp83. This promotes Hsp83 binding to both the PAS-B and bHLH domains of the Met protein. Subsequently, Hsp83 interacts with the nuclear pore protein Nup358. Under the guidance of the nuclear pore complex, the JH-Met-Hsp83 protein complex traverses the nuclear pore, entering the nucleus (He et al., 2014; Gao et al., 2022a). bHLH-PAS family proteins typically require pairing with other co-regulatory factors to exert transcriptional functions. Thus, as an intracellular receptor, Met’s transcriptional activity may also necessitate association with co-regulatory factors. In *Aedes aegypti*, yeast two-hybrid screening identified the steroid receptor coactivator βFtz-F1-Interacting Steroid receptor Coactivator (FISC) as a co-transcription factor that binds to Met, which also belongs to the bHLH-PAS family of proteins (Zhu et al., 2006; Li et al., 2010). When FISC lacks the PAS-A and PAS-B domains, it no longer binds to Met (Li et al., 2010). In the common house cricket, the homolog of FISC is Steroid Receptor Coactivator (SRC) (Zhang et al., 2011), and successful binding of JH to the PAS-B domain of Met is a prerequisite for Met-SRC interaction (Charles et al., 2011). In *D. melanogaster*, the homolog of FISC/SRC is Taiman (Tai), with Met binding Tai via its PAS domain (Charles et al., 2011; Lozano and Belles, 2011; Tumova and Jindra, 2023).

As the intracellular receptor for JH, Met functions as a transcription factor, initiating transcriptional expression of downstream genes after entering the nucleus. In *D. melanogaster*, the JH-Met-Tai-Hsp83 complex binds to the JH response element (JHRE) within the promoter region of the JH primary response gene *Krüppel homolog 1* (*Kr-h1*), thereby activating *Kr-h1* transcription (Wilson and Fabian, 1986; Jindra et al., 2024). In the silkworm, JH enters the cell and forms a complex with Met2 and SRC, directly inducing *Kr-h1* transcription. The JHRE on the *Kr-h1* promoter, which contains the E-box sequence (CACGTG), is crucial for the strong induction of *Kr-h1* transcription levels (Kayukawa et al., 2012). In *Ae. aegypti*, another JH primary response gene, *Early trypsin* (*ET*), has been identified. In females, when JH titers are high, the JH-Met-FISC complex binds to the JHRE on the *ET* gene promoter, initiating its transcriptional expression to prepare for blood feeding and yolk development (Li et al., 2010).

### 2.4 Molecular mechanism of JH-Met/Gce-Kr-h1

As a primary response gene to intracellular JH signaling, *Kr-h1* encodes a key factor antagonizing insect metamorphosis (Konopova et al., 2011; Lozano and Belles, 2011). As early as 1986, the zinc finger domain-containing transcription factor protein Krüppel (Kr) was discovered in *D. melanogaster*, along with multiple homologous proteins, including Kr-h1 (Schuh et al., 1986). Subsequent studies revealed *Kr-h1*’s role in antagonizing insect metamorphosis (Jindra et al., 2013; Cheng et al., 2025; Wu et al., 2025). Studies in *D. melanogaster* reveal that the *Kr-h1* gene encodes a DNA-binding transcription factor protein with eight C2H2 zinc finger structures. The primary transcript of the *Kr-h1* gene undergoes alternative splicing to yield three distinct N-terminal sequence isoforms: *Kr-h1α*, *Kr-h1β*, and *Kr-h1γ*. Since *Kr-h1α* and *Kr-h1γ* are difficult to distinguish and *Kr-h1α* expression is tenfold higher than *Kr-h1γ*, both are collectively termed *Kr-h1α (*Minakuchi et al., 2008). Kr-h1β possesses the longest amino acid sequence, with its protein N-terminal sequence exceeding that of Kr-h1α by 54 amino acids. *Kr-h1α* exhibits high expression primarily during the larval, prepupal, and adult stages. Mutations in *Kr-h1α* cause pupal lethality in *D. melanogaster*, indicating its role in regulating insect pupal metamorphosis (Pecasse et al., 2000; Beck et al., 2004; He et al., 2020). In contrast, *Kr-h1β* is mainly expressed during the embryonic stage, primarily influencing embryonic metamorphosis and nervous system formation (Beck et al., 2004).

### 2.5 JH intracellular signaling antagonizes 20E signaling

JH antagonizes 20E signaling to prevent precocious metamorphosis during the larval stages, by inducing Kr-h1 expression, thereby inhibiting metamorphosis regulation (Liu S. et al., 2018). The JH-20E synergistic regulation of metamorphosis involves three key genes: *Kr-h1*, *Broad Complex* (*Br-C*), and *Ecdysone-induced protein 93* (*E93*). In holometabolous insects, Br-C promotes pupal stage formation. 20E directly induces *Br-C* expression, while Kr-h1 suppresses *Br-C* expression to prevent premature metamorphosis in larvae (Zhou et al., 1998; Zhou and Riddiford, 2002; Urena et al., 2014). During the larval stage of hemimetabolous insects, JH promotes *Br-C* expression by activating *Kr-h1* transcription, and Br-C facilitates wing primordium development (Erezyilmaz et al., 2006; Huang et al., 2013). E93 serves as a key initiator of metamorphosis and a crucial factor for adult formation; Kr-h1 antagonizes 20E signaling by directly suppressing *E93* expression (Kayukawa et al., 2017). In the hemimetabolous *Blattella germanica*, RNA interference targeting *E93* induces the emergence of over-aged nymphs. Conversely, in holometabolous insects like *T. Castaneum* and *D. melanogaster*, it causes secondary pupation and pupal mortality (Urena et al., 2014). Alternatively, JH inhibits ecdysis by suppressing 20E synthesis via Kr-h1. As early as 1989, studies on silkworm larvae revealed that JH inhibits ecdysone secretion in the prothorax (Sakurai et al., 1989). JH suppresses the expression of ecdysone synthesis-related Halloween genes—such as *Spook*, *Phantom*, *Disembodied*, and *Shadow*—via Kr-h1 (Yamanaka et al., 2007; Ogihara et al., 2015; Liu S. et al., 2018). Kr-h1 directly binds to the upstream binding sites (KBS) of the *Spookier* gene, inducing methylation and thereby suppressing *Spookier* gene expression (Zhang et al., 2018).

### 2.6 Intracellular JH signaling promotes reproduction

JH influences insect reproduction through intracellular signaling pathways. In adult *Locusta migratoria*, JH directly activates *Kr-h1* transcription via the intracellular receptor Met, inducing *vitellogenin* (*Vg*) expression in the fat body to promote ovarian development and lipid accumulation. Concurrently, JH activates the transcriptional expression of *Minichromosome Maintenance proteins 4* and *7* (*Mcm4*/*Mcm7*) and *cell division cyclin 6* (*Cdc6*), promoting DNA replication and polyploidy in fat body cells. This facilitates the substantial transcriptional expression of reproductive-related proteins, including *Vg (*Guo et al., 2014; Song et al., 2014; Wu et al., 2016). In *D. melanogaster*, JH induces *collagen IV* gene expression in adult fat body cells via intracellular receptors. Collagen IV protein participates in assembling the extracellular matrix (ECM) of ovarian muscle cells, and ECM integrity ensures proper ovulation. In *Met^27^*(*Met* mutant) and *Gce^2.5K^* (*Gce* mutant) *D. melanogaster* strains, where intracellular signaling is weakly blocked, mature eggs accumulate in the ovaries, causing ovarian enlargement and abdominal distension in female flies (Luo et al., 2021).

## 3 Juvenile hormone membrane signaling

### 3.1 Discovery of juvenile hormone membrane signaling

During JH research in the last century, scientists not only discovered that JH maintains the larval state of insects via intracellular receptors; they also observed that JH rapidly induces physiological responses across multiple insect species. In 1974, Davey and Huebner observed in *R. prolixus* that JH treatment caused rapid shrinkage of the follicular epithelial cells surrounding the oocytes, creating large gaps between cells (Davey and Huebner, 1974). Using ^3^H-labeled JH I for detection, it was found that JH I binds specifically to follicular cell membranes but not to brain cell membranes. Furthermore, JH I binds to cell membranes in a specific, saturable manner with Kd value of 6.54 nM (Ilenchuk and Davey, 1985). This experimental finding suggests that JH promotes Vg absorption by binding to a specific membrane protein.

Similar regulatory mechanisms exist in *L. migratoria* and *Heliothis virescens*, where JH similarly induces the “Patency” phenomenon in follicular cells (Davey et al., 1993; Pszczolkowski et al., 2008). In *L. migratoria*, incubation of follicular cell soluble membrane proteins with [^3^H]-EFDA (a photoreactive analog of JH III) detected binding to a 35 kDa polypeptide. Pretreatment of membrane proteins with JH III blocked [^3^H]-EFDA binding to this protein. [^3^H]-EFDA bound to this membrane-extracted protein in a specific, saturable manner with a Kd of 3.68 nM. Unlike the follicular cells of the *R. prolixus*, *L. migratoriat* follicular cells did not respond to JH I. In *L. migratoria*, JH II also induces volume reduction in follicular cells. Incubation of cell membrane extracts with [^3^H]-EHDA (a photoreceptor analog of JH II) revealed that [^3^H]-EHDA non-specifically binds to the 35 kDa peptide. Conversely, MKD (a photoreceptor analog of methoprene) binds to a 17 kDa peptide. Incubation of brain and fat body membrane extracts with [^3^H]-EFDA revealed binding to 70 kDa and 58 kDa peptides, respectively. This indicates that these JH target organs may partially respond to JH via membrane receptors or possess a membrane-mediated JH uptake mechanism (Sevala et al., 1994).

The male accessory gland of *D. melanogaster* serves as the seminal vesicle synthesis and secretion organ within the male reproductive system, playing a crucial role in female fertility and male fecundity. It primarily secretes reproductive-associated proteins, such as sex peptides and exosomes, collectively termed seminal proteins (Chapman et al., 2003). JH rapidly induces protein synthesis in male *D. melanogaster* accessory glands, which modulate reproductive capacity in response to JH signaling. Additionally, *in vitro* treatment of larval salivary glands with JH rapidly induces mitochondrial ultrastructural changes, a phenomenon independent of transcriptional and translational regulation (Yamamoto et al., 1988; Farkas and Sutakova, 2001). In 1995, Sevala demonstrated the presence of JH photoreceptor ligand-binding proteins in membrane extracts from male accessory glands of the *R. prolixus* and *L. migratoria*. [^3^H]-EBDA (a photoreceptor analog of JH I) binds to a roughly 51 kDa peptide in membrane extracts from male accessory gland cells of the *R. prolixus*. [^3^H]-EFDA (a light-affinity analog of JH III) bound to a polypeptide of approximately 35 kDa in cell membrane extracts from the male accessory glands of *L. migratoria (*Wyatt and Davey, 1996).

### 3.2 Potential membrane receptors for juvenile hormone

#### 3.2.1 G protein-coupled receptors

The synthesis of vitellogenin and its uptake by maturing oocytes during egg maturation are essential for successful female reproduction. Ecdysteroids control this process in Diptera and some Hymenoptera and Lepidoptera. In Diptera, such as *Ae. aegypti*, vitellogenesis is regulated by blood meal-triggered pathways that are responsible for transcription of the *Vg* gene, with 20E signaling being the principal regulator (Roy et al., 2018). But in most insects, Juvenile hormones act as gonadotropins, regulating vitellogenesis. The induction of “patency” by JH in *R. prolixus* is remarkably rapid, reversible, and unaffected by macromolecular synthesis inhibitors, indicating that this process does not involve gene transcription, translation, or protein synthesis (Davey and Huebner, 1974; Abu-Hakima and Davey, 1977). Subsequent studies demonstrated that *in vitro* treatment of follicular cells with JH increases the activity of Na^+^/K^+^-ATPase on the follicular cell membrane. The resulting change in cellular osmotic pressure leads to a reduction in cell volume and the appearance of the “Patency” phenomenon, facilitating the entry of Vg proteins into the oocyte. Furthermore, adding the Na^+^/K^+^-ATPase inhibitor ouabain during JH treatment suppressed the JH-induced “patency” phenomenon (Ilenchuk and Davey, 1982). Protein Kinase C (PKC) also participates in JH-induced Na^+^/K^+^-ATPase activation, acting upstream of the enzyme. Similarly, PKC inhibitor treatment suppresses JH effects, while PKC activator treatment mimics JH action—a process blocked by ouabain (Sevala and Davey, 1988). JH treatment increases the phosphorylation level of a 100 kDa peptide (belonging to an α-subunit polypeptide of Na^+^/K^+^-ATPase) in follicular cells. This increased phosphorylation can also be induced by PKC activators, inhibited by PKC inhibitors, and blocked by ouabain (Davey, 1996).

Subsequent experiments in the Orthopteran insect, *L. migratoria*, demonstrated that JH activates a protein network involving G protein-coupled receptors (GPCRs), receptor tyrosine kinases (RTKs), phospholipase C (PLC), inositol trisphosphate receptor (IP3R), and PKC. This leads to phosphorylation of Ser8 in the Na^+^/K^+^-ATPase α-subunit, thereby enhancing Na^+^/K^+^-ATPase activity and inducing “patency” (Jing et al., 2018). JH also activates the membrane signaling pathway GPCR-PLC-PKC to phosphorylate the Vitellogenin receptor (VgR). Phosphorylation of VgR is essential for its translocation from the oocyte cytoplasm to the membrane. Phosphorylated VgR moves to the cell membrane, binds to Vg, and promotes oocyte uptake of Vg (Jing et al., 2021). To identify the specific molecular nature of JH membrane receptors, GPCR screening experiments were conducted in *L. migratoria*. Twenty-two GPCRs were identified in the ovarian transcriptome, with 21 also expressed in the fat body. RNAi screening for all identified GPCRs showed that injection of *LGR4* and *Oct*/*TyrR* dsRNA caused reduction of *LGR4* or *Oct*/*TyrR* mRNA levels in the fat body and ovary, respectively. Western blotting indicated that Vg protein levels decreased in the fat body. These observations suggest that LGR4 and Oct/TyrR may influence Vg protein synthesis in the fat body. Injection of *mAChR-C*, *OR-A1*, *OR-A2*, and *CirlL* dsRNA led to reduction of their mRNA levels in the fat body and ovary, respectively. *Vg* mRNA levels remained unchanged, and Vg protein expression was either unchanged or increased, but decreased in the hemolymph or ovary. In addition, ovarian growth and oocyte maturation were inhibited. Therefore, OR-A1, OR-A2, mAChR-C, and CirlL regulate Vg transportation and uptake. However, the specific JH membrane receptor protein(s) remains unidentified (Zheng et al., 2021).

In the Lepidopteran insect, *H. virescens*, the emergence of “patency” is regulated by JH II and JH III through PKC-dependent calcium channels, which activate Na^+^K^+^-ATPase activity. Conversely, JH I induces “patency” via the cAMP second messenger system (Pszczolkowski et al., 2008). Further studies on *H. virescens* revealed that GPAnt-2 (a peptide antagonistic to GPCRs) inhibits JH I-induced “Patency”, leading researchers to hypothesize that JH I-mediated “Patency” in this species also functions via GPCRs (Pszczolkowski et al., 2005).

In the Lepidopteran insect, *Helicoverpa armigera*, the *Broad isoform Z7* (*BrZ7*) was found to be lowly expressed during larval growth, with the BrZ7 protein in a phosphorylated state. However, during metamorphosis, the gene was highly expressed while the protein remained unphosphorylated. Knockout of *BrZ7* in larvae and *in vitro* JH treatment both inhibit expression of 20E-responsive genes, preventing metamorphosis. Phosphorylation of BrZ7 protein is induced by JH activation of GPCR, PLC and PKC. Phosphorylated BrZ7 then suppresses 20E signaling by regulating *Calponin* gene expression (Cai et al., 2014).

To identify putative membrane receptors GPCRs, researchers screened two rhodopsin receptors and 111 non-sensory GPCRs in *T. castaneum*, including 74 rhodopsin-like GPCRs, 19 secretin-like GPCRs, 11 metabolic glutamate-like GPCRs, and 7 atypical GPCRs, using RNAi technology. This screening identified 41 GPCRs critically affecting female reproduction, with only two GPCRs (Rhodopsin-like receptor and Dopamine D2-like receptor) being essential for Vg uptake (Bai and Palli, 2016).

In insects such as Orthoptera, Lepidoptera, and Coleoptera, it has been demonstrated that JH activates PKC and Na^+^K^+^-ATPase through potential membrane receptors GPCRs, inducing the “patency” phenomenon and promoting Vg uptake in oocytes (Yang et al., 2018). GPCRs belong to the transmembrane receptor protein family, possessing seven transmembrane helices and thus also termed seven-transmembrane receptors. Their structure includes an extracellular N-terminus, an intracellular C-terminus, and three extracellular loops linked by transmembrane helices. GPCRs can be activated by hormones or neurotransmitters, subsequently activating downstream signaling molecules within cells to produce corresponding physiological responses (Rosenbaum et al., 2009). However, comprehensive screening experiments for GPCRs in *T. castaneum* and *L. migratoria* have failed to identify JH membrane receptors.

#### 3.2.2 Receptor tyrosine kinases

In 1988, Yamamoto et al. discovered in *D. melanogaster* that *in vitro* treatment of male accessory glands with 0.01–1 nM JH III increased both total RNA and protein synthesis in the glands. Ca²^+^ was essential for this effect: JH III promotion was weakened in the absence of Ca²^+^, and its action disappeared when Ca²^+^ was chelated. The functional Ca²^+^ concentration required for JH III *in vitro* was 0.1 mM. Higher Ca²^+^ concentrations did not affect glandular JH responsiveness but could enhance protein synthesis in the absence of JH. The presence of Co²^+^ (a Ca²^+^ antagonist) attenuated the Ca²^+^ effect, whereas adding other second messengers like cyclic AMP did not influence JH-stimulated protein synthesis. These results indicate Ca²^+^ participation in JH-stimulated protein synthesis. Researchers also discovered PKC involvement in the rapid JH response pathway within the accessory gland. PDBU, a PKC activator, promotes male accessory gland protein synthesis in Ca²^+^-containing *in vitro* culture media. When normal male accessory glands were treated with Methoprene *in vitro*, increased glandular protein synthesis was detectable within just one hour. However, when the same treatment was applied to the male accessory glands of the mutant fruit fly *tur* (lacking PKC activity), no increase in protein synthesis was observed (Yamamoto et al., 1988).

In the central nervous system of the silkworm, JH I can induce phosphorylation of an approximately 48 kDa protein via RTK, a process partially dependent on calcium ions (Arif et al., 2002). Experimental results from the East Asian locust also indicate that RTK may participate in JH-induced “patency” (Jing et al., 2018). In the Diptera insect, *Ae. aegypti*, researchers observed that JH rapidly increases intracellular levels of inositol 1,4,5-trisphosphate (IP_3_), diacylglycerol (DAG), and calcium ions, indicating JH activates the PLC pathway (Liu et al., 2015). The PLC pathway is typically activated by cell membrane receptors (GPCRs and RTKs), leading to the hydrolysis of phosphatidylinositol 4,5-bisphosphate (PIP_2_) on the cell membrane to form IP_3_ and DAG. IP3 diffuses to the endoplasmic reticulum (ER), binds its receptor IP_3_R, and releases Ca²^+^ stored in the ER into the cytoplasm (Vines, 2012; Emmanouilidi et al., 2017). During JH-induced calcium upregulation, treatment with different inhibitors revealed that revealed that GPCR inhibitors (Suramin and GDP-β-S) failed to block JH-induced calcium ion concentration elevation, whereas RTK inhibitors (Genistein and Tyrphostin A23) completely blocked this response. The elevated calcium ion concentration activates Calcium/Calmodulin-dependent protein Kinase II (CaMKII) (Liu et al., 2015). Additionally, JH activates PLC via a membrane signaling pathway, which further activates PKC (Ojani et al., 2016). In the fat body of adult *Ae. aegypti* following emergence, JH induces selective splicing of Tai by activating the RTK, Phosphatidylinositide 3-kinase (PI3K), and Protein kinase B (AKT) pathways (Liu P. et al., 2018). In *D. melanogaster*, experiments including phosphoproteomics, genetics, and inhibitor treatments validated that JH activates phosphorylation of serine 35 in USP, one of the 20E heterodimeric receptors, via the RTK-PLC-PKC Pathway (Gao et al., 2022b). These results demonstrate that JH membrane signaling activates the RTK-PLC-PKC-CaMKII pathway; and this pathway is conserved in Diptera insects (*Ae. aegypti* and *D. melanogaster*) (Liu et al., 2015; Ojani et al., 2016). Consequently, Researchers hypothesize that the JH membrane receptor in Diptera may be an RTK.

Recent advances have significantly expanded our understanding of the role of RTKs in JH signaling. In 2025, a study using *Ae. aegypti* identified receptor tyrosine kinase PVR (PDGF/VEGF-receptor related) - homologous to mammalian platelet-derived growth factor receptors (PDGFR) and vascular endothelial growth factor receptors (VEGFR) - as a key mediator of membrane-initiated JH signaling (Zhao et al., 2025). Concurrently, using the major agricultural pest *H. armigera* as a model, microcalorimetry (MST) and isothermal titration calorimetry (ITC) techniques confirmed that RTKs CAD96CA and FGFR1 exhibit significantly stronger binding affinity to JH III than other JH analogues. Knockout experiments using CRISPR/Cas9 gene editing revealed that larvae lacking these receptors pupated prematurely, exhibited significantly reduced expression of the JH downstream gene *Kr-h1*, and showed activation of ecdysone pathway genes. Knockdown of *CAD96CA* and *FGFR1* homologs in *Spodoptera frugiperda* (Sf9 cells) and *D. melanogaster* (S2 cells) similarly blocked JH-induced calcium signaling and gene expression, indicating the conservation of this receptor as a cellular membrane receptor for juvenile hormones in insects. Through systematic screening and detailed experiments, two JH membrane receptors—CAD96CA and FGFR1—were first identified (Li et al., 2025). However, since the natural hormones of Lepidoptera larvae are JH I and JH II, whether CAD96CA and FGFR1 bind to JHs other than JH III (JH I and JH II) remains to be addressed in future studies.

Extensive evidence now confirms the existence of JH membrane receptors, suggesting they may function as GPCRs or RTKs. These receptors activate downstream protein phosphorylation cascades, triggering diverse physiological responses. To date, only two JH III membrane receptors have been identified in *H. armigera*. Regulatory patterns of JH membrane receptors may vary across insect species or tissues, and the precise molecular nature of these receptors remains a key research focus in the JH field.

### 3.3 JH membrane signaling promotes intracellular signaling

JH membrane signaling triggers a cascade of protein kinase reactions involving multiple kinases. JH intracellular receptors Met and other intracellular signaling components are also phospho-modulable proteins. JH activates protein kinases via membrane signaling, leading to the phosphorylation of multiple intracellular signaling components and thereby promoting intracellular JH signaling. In *Ae. aegypti*, JH membrane signaling activates PLC, PKC, and CaMKII, which in turn phosphorylate Met and Tai, thereby promoting the JH intracellular receptor signaling pathway. Notably, the activation of PKC by JH membrane signaling is essential for Met binding to JHRE (Liu et al., 2015; Ojani et al., 2016). Additionally, in *Ae. aegypti* Aag-2 cells, JH treatment was found to induce phosphorylation of Met at serine 77 and 710, while dephosphorylating at serine 73 and 747. Within 1 h after treatment, JH promoted phosphorylation at both threonine 664 and serine 723. Intriguingly, 24 h after JH treatment, the JH-dependent phosphorylation at threonine 664 and serine 723 were reconstituted. Furthermore, JH induces dephosphorylation of serine 694 within the evolutionarily conserved SVIQ motif of Kr-h1. The Kr-h1 dephosphorylation mutant (S694V) showed significantly higher activity in inducing the luciferase gene regulated by JH response elements (Liu et al., 2015; Kim et al., 2021). In *H. armigera*, JH promotes phosphorylation of Met1 at serine 393 of the PAS-B domain, which is crucial for Met1 binding to the JHRE in the Kr-h1 promoter. Phosphorylation at this site also enhances the interaction between Met1 and Tai (Li et al., 2021). In *D. melanogaster*, JH induces phosphorylation of serine 55 and threonine 76 in the bHLH domain of Met protein via membrane receptors, thereby promoting expression of downstream target gene *Kr-h1*. Concurrently, the JH membrane receptor triggers phosphorylation of serine 219 in chaperone Hsp83, thereby enhancing Hsp83-Met binding to assist the nuclear translocation of the intracellular receptor Met and facilitate intracellular JH signaling (Gao et al., 2022a).

### 3.4 JH membrane signaling promotes 20E signaling

In *D. melanogaster*, JH activates the PLC-PKC signaling pathway via the membrane receptor RTK, causing phosphorylation at serine 35 of USP, one of the heterodimeric receptors for 20E. Using CRISPR/Cas9 technology, we generated the USP Ser35 mutant fly line *usp^S35A^*. Compared to wild-type flies, these mutant flies exhibited delayed pupation by approximately 6 hours (Wang et al., 2013; Gao et al., 2022b). Further analysis revealed reduced transcription of the Halloween gene (ecdysone synthase) in the *usp^S35A^* strain, leading to overall diminished 20E signaling. This manifested as significantly lower mRNA expression of primary response genes *Br-C* and *E75*, thereby causing delayed pupation (Gao et al., 2022b). In the fat body of adult *Ae. aegypti* following emergence, four distinct isoforms of *Tai* exist: *Tai-A*, *Tai-B*, *Tai-C*, and *Tai-D*. JH induces selective splicing of Tai by activating the RTK-PI3K-AKT pathway, increasing the production of *Tai A/B* variants while reducing the formation of *Tai C/D* variants. Compared to Tai C/D, Tai A/B isoforms exhibit stronger binding affinity to the 20E receptor complex. In contrast, all four isoforms exhibit similar binding affinity to the JH receptor complex. Pre-ovogenetic induction of *Tai A/B* isoform production by JH is essential for the 20E-regulated cascade of events in blood-feeding-initiated oocyte maturation in *Ae. aegypti* (Liu P. et al., 2018).

## 4 Summary and discussion

JH is one of the most critical developmental regulatory hormones in insects. Experimental studies across various insect species have demonstrated that JH participates in regulating complex physiological processes through distinct signaling pathways. Both intracellular and membrane-bound JH signaling play indispensable roles in insect metamorphosis and reproduction, yet numerous questions remain to be explored by researchers. Existing data demonstrate that JH intracellular signaling antagonizes 20E signaling, while JH membrane signaling promotes both 20E signaling and ecdysone synthesis. However, the molecular mechanisms underlying these effects remain poorly understood and warrant further investigation. JH membrane signaling promotes intracellular JH signaling by phosphorylating multiple protein components within intracellular signaling pathways. However, detailed elucidation of the numerous proteins and protein kinases involved in this process remains necessary. Additionally, elucidating the JH membrane signal-activated protein kinase cascade and its associated kinases could explain JH-induced protein phosphorylation mechanisms. However, research on how JH membrane signals inhibit protein phosphorylation and induce dephosphorylation remains largely unexplored. Identifying the specific molecular nature of JH membrane receptors stands as one of the most challenging current research areas. Advances in biotechnology are expected to yield breakthroughs in this field. Is the membrane receptor conserved across different species? Are the membrane receptors present in Diptera identical to those in Orthoptera, Coleoptera, and Lepidoptera? Is JH membrane signaling activated only in specific tissues? A deeper and more comprehensive understanding of the molecular mechanisms by which JH membrane signaling regulates insect growth and development remains to be explored.

## Data Availability

The original contributions presented in the study are included in the article/supplementary material. Further inquiries can be directed to the corresponding author.
